# Diagnostic and Prognostic Value of *B4GALT1* Hypermethylation and Its Clinical Significance as a Novel Circulating Cell-Free DNA Biomarker in Colorectal Cancer

**DOI:** 10.3390/cancers11101598

**Published:** 2019-10-19

**Authors:** Francesco Picardo, Antonella Romanelli, Laura Muinelo-Romay, Tommaso Mazza, Caterina Fusilli, Paola Parrella, Jorge Barbazán, Rafael Lopez-López, Raffaela Barbano, Mariangela De Robertis, Chiara Taffon, Veronica Bordoni, Chiara Agrati, Manuela Costantini, Francesca Ricci, Paolo Graziano, Evaristo Maiello, Lucia Anna Muscarella, Vito Michele Fazio, Maria Luana Poeta

**Affiliations:** 1 Laboratory of Molecular Medicine and Biotechnology, University Campus Bio-Medico of Rome, 00128 Rome, Italy; f.picardo@unicampus.it (F.P.); fazio@unicampus.it (V.M.F.); 2 Department of Bioscience, Biotechnology and Biopharmaceutics, University of Bari, 70126 Bari, Italy; romanelli.antonella@gmail.com (A.R.); m.costantini@unicampus.it (M.C.); 3 Liquid Biopsy Analysis Unit, Translational Medical Oncology Group (Oncomet), CIBERONC, Health Research Institute of Santiago de Compostela (IDIS), University Hospital of Santiago de Compostela (SERGAS), 15706 Santiago de Compostela, Spain; laura.muinelo.romay@sergas.es (L.M.-R.); jorgebarbazan@gmail.com (J.B.); rafael.lopez.lopez@sergas.es (R.L.-L.); 4 Unit of Bioinformatics, Fondazione IRCCS “Casa Sollievo della Sofferenza”, 71013 San Giovanni Rotondo (Fg), Italy; t.mazza@css-mendel.it (T.M.); caterinafusilli@gmail.com (C.F.); 5 Laboratory of Oncology, Fondazione IRCCS “Casa Sollievo della Sofferenza”, 71013 San Giovanni Rotondo (Fg), Italy; pparrella@operapadrepio.it (P.P.); r.barbano@operapadrepio.it (R.B.); l.muscarella@operapadrepio.it (L.A.M.); 6 Institut Curie, PSL Research University, F-75005 Paris, France; 7 Institute of Biomembranes, Bioenergetics and Molecular Biotechnologies, Consiglio Nazionale delle Ricerche (CNR), 70126 Bari, Italy; mariangela.derobertis@gmail.com; 8 Unit of Pathology, University Campus Bio-Medico of Rome, 00128 Rome, Italy; c.taffon@unicampus.it; 9 Cellular Immunology and Pharmacology Laboratory, “Lazzaro Spallanzani” National Institute for Infectious Diseases, Istituto di Ricovero e Cura a Carattere Scientifico (IRCCS), 00149 Rome, Italy; veronica.bordoni@inmi.it (V.B.); chiara.agrati@inmi.it (C.A.); 10 Department of Urology, IRCCS-Regina Elena National Cancer Institute, 00144 Rome, Italy; 11 Laboratory of Pathology, Istituto Dermopatico dell’Immacolata IDI-IRCCS, 00167 Rome, Italy; francesca.ricci@idi.it; 12 Pathology Unit, Fondazione IRCCS “Casa Sollievo della Sofferenza”, 71013 San Giovanni Rotondo (Fg), Italy; p.graziano@operapadrepio.it; 13 Department of Oncology, Fondazione IRCCS “Casa Sollievo della Sofferenza”, 71013 San Giovanni Rotondo (Fg), Italy; e.maiello@operapadrepio.it

**Keywords:** *B4GALT1*, colorectal cancer, predictive biomarker, cetuximab, liquid biopsy, cfDNA

## Abstract

Epigenetic modifications of glyco-genes have been documented in different types of cancer and are tightly linked to proliferation, invasiveness, metastasis, and drug resistance. This study aims to investigate the diagnostic, prognostic, and therapy-response predictive value of the glyco-gene *B4GALT1* in colorectal cancer (CRC) patients. A Kaplan–Meier analysis was conducted in 1418 CRC patients (GEO and TCGA datasets) to assess the prognostic and therapy-response predictive values of the aberrant expression and methylation status of *B4GALT1*. Quantitative methylation-specific PCR (QMSP) and droplet digital quantitative methylation-specific PCR (dd-QMSP) were respectively used to detect hypermethylated *B4GALT1* in metastasis and plasma in four cohorts of metastatic CRC cases (mCRC). Both the downregulated expression and promoter hypermethylation of *B4GALT1* have a negative prognostic impact on CRC. Interestingly a low expression level of *B4GALT1* was significantly associated with poor cetuximab response (progression-free survival (PFS) *p* = 0.01) particularly in wild-type (WT)*-KRAS* patients (*p* = 0.03). *B4GALT1* promoter was aberrantly methylated in liver and lung metastases. The detection of hypermethylated *B4GALT1* in plasma of mCRC patients showed a highly discriminative receiver operating characteristic (ROC) curve profile (area under curve (AUC) value 0.750; 95% CI: 0.592–0.908, *p* = 0.008), clearly distinguishing mCRC patients from healthy controls. Based on an optimal cut-off value defined by the ROC analysis, *B4GALT1* yield a 100% specificity and a 50% sensitivity. These data support the potential value of *B4GALT1* as an additional novel biomarker for the prediction of cetuximab response, and as a specific and sensitive diagnostic circulating biomarker that can be detected in CRC.

## 1. Introduction

Colorectal cancer (CRC) is the fourth most commonly diagnosed cancer considering together males and females, with over 1.7 million new cases and 861,663 deaths estimated for 2018 worldwide [1]. It is well established that the prognosis of patients is associated with the tumor stage and despite the continuous and recent advancements in diagnosis and treatment more than 50% of CRCs metastasize to lymph nodes, and distant organs such as the liver and lung, and the mean five year survival rate of metastatic CRC (mCRC) is estimated to be still low (~10%) [1,2].

Tumor progression is indeed one of the most critical factors that impact CRC prognosis. Current approaches which rely on the combination of radiologic examinations and serum protein biomarkers measurements are routinely used for the detection and the monitoring of disease progression. However these tests have limited sensitivity and specificity [3,4,5]. Emerging evidences have shown that the detection of circulating tumor DNA (ctDNA) may represent a minimally-invasive strategy for the early prediction of tumor progression before the clinical appearance of metastasis [6,7]. From this perspective, in recent years a great interest has been raised to identify novel diagnostic, prognostic, and therapy-response predictive molecular biomarkers such as those tested in tumor tissue as well as in metastases and in the bloodstream [8,9]. A plethora of molecules (protein, DNA, RNA) and CTC–based assays (Circulating Tumor Cell) have been used for CRC diagnosis, staging, prognosis, and therapy-response prediction [6,10,11].

It is well established that both genetic and epigenetic alterations play a key role in the initiation and progression of colorectal carcinogenesis, and that they can be recognized in the serum or plasma DNA of cancer patients [12,13,14]. One of the most frequent epigenetic alterations is DNA methylation which leads to the silencing of gene expression and regulates different cell functions, such as, among others, proliferation, apoptosis, DNA repair, metabolism, and angiogenesis [15,16,17]. In contrast to mutation detection, which requires a patient-specific assay designed to cover the whole spectrum of gene mutations, methylation analysis can circumvent this limit since it is often restricted to defined genomic loci such as the promoter region. Several genes have been found to be hypermethylated in different type of cancers including CRC [18,19], and some of them have been recently proposed as circulating biomarkers [7,20]. Interestingly the up or down regulation of glyco-genes has been well documented in different tumor types, and their related pathways play a crucial role in the regulation of cell growth, invasiveness, metastasis, and drug resistance [21,22]. Only a few studies explored the epigenetic mechanisms underlying the aberrant expression of glycan genes in cancer cells [23,24,25]. In this study we investigated the expression and methylation status of beta-1,4-galactosyltransferase 1 (*B4GALT1*) in different cohorts of CRC. This glycoprotein is a type II membrane-bound glycoprotein that transfers UDP-galactose to different sugar acceptors as N-acetylglucosamine (GlcNAc) [26,27]. Furthermore, *B4GALT1* interacts directly with *EGFR* inhibiting the dimerization of the receptor and the tyrosine phosphorylation and its activation in human hepatocellular carcinoma cells, suggesting an inhibitory role of *B4GALT1* in *EGFR* signaling pathway [28,29].

In our previous studies [30,31] we demonstrated that the glyco-gene *B4GALT1* expression is downregulated, through hypermethylation, in primary tumors of CRC patients yielding a specificity of 91.7% and a sensitivity of 54% and that it seems to characterize the invasive phenotype of adenocarcinoma, whereas it is significantly less frequently methylated in early lesions such as adenoma. In the present study we investigated the prognostic and cetuximab-response prediction significance of aberrant methylation and the expression status of *B4GALT1* in 1418 CRC patients from multiple cohorts of the Gene Expression Omnibus (GEO) public data repository and The Cancer Genome Atlas (TCGA) database. Furthermore we determined the methylation status of *B4GALT1* in metastatic lesions of CRC (Group 1 and 2, *n* = 49 cases). For the first time we also assessed whether the hypermethylation of *B4GALT1* could be a useful potential diagnostic biomarker in the blood compartment, as detectable in cell-free DNA (cfDNA) of mCRC plasma samples. To accomplish this we indeed analyzed the tissue and plasma samples of training and validation sets of mCRC patients (Group 3T and 3V, *n* = 51 cases) showing that this biomarker is able to distinguish metastatic cases from healthy control subjects.

## 2. Materials and Methods

### 2.1. Sample Collection

**Figure 1:**
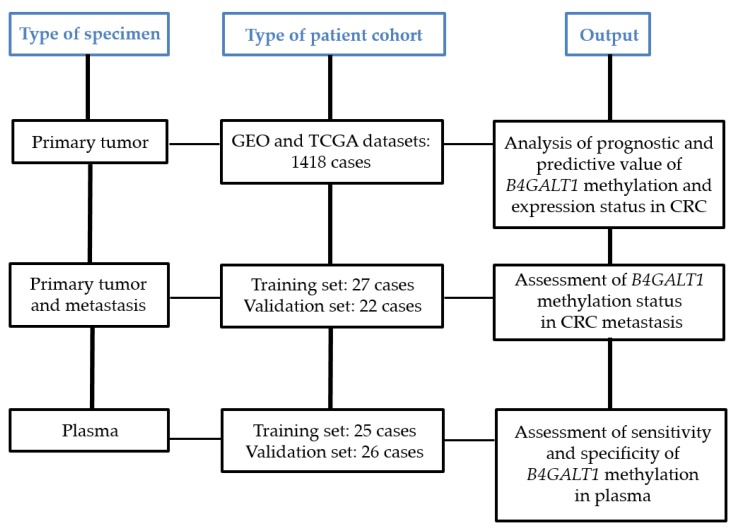
Study design and sample selection workflow.

Different types of sample cohorts and specimens have been used for the present study (Figure 1). In particular, we analyzed: (a) primary tumors of 1418 cases derived from TGCA (total *n* = 300) and GEO datasets (total *n* = 1118); (b) primary tumors, paired normal mucosa, metastases derived from a training and validation set of 49 cases collected at University Campus Bio-Medico of Rome (UCMB Group 1 training-set, *n* = 27 patients), IRCCS “Casa Sollievo della Sofferenza” (IRCCS-CSS Group 2 validation-set, *n* = 22 patients); (c) primary tumors and plasmas of a training and validation set of 51 CRC and 19 healthy donors collected respectively at University Hospital of Santiago de Compostela (CHUS, Biobank PT17/0015/0002, integrated in the Spanish National Biobanks Network, Group 3T training-set, *n* = 25 patients, and 3V validation-set, *n* = 26 patients), Lazzaro Spallanzani National Institute for Infectious Diseases (INMI, Group 4, *n* = 19 healthy donors) (Table S1). For the formalin fixed paraffin embedded (FFPE) specimens, colorectal tumor, metastasis, and normal colorectal mucosa samples of 5 μm sections were cut from the primary tumor specimens for hematoxylin-eosin staining to inspect the presence of neoplastic cells and to ensure the absence of neoplastic cells in normal mucosa. Additional 10 μm sections were cut for DNA extraction. Peripheral blood samples from 25 (training set Group 3T) and 26 (validation set Group 3V) patients with mCRC were collected at CHUS from July 2008 to June 2016. Peripheral blood samples were drawn using 10 mL EDTA tubes (Vacutainer, Becton Dickinson, New Jersey, USA) and plasma was separated by centrifugation at 1750× *g* for 10 min and stored at −80 °C until use. In addition, plasma samples (negative controls) from 19 healthy volunteers were collected at the INMI and at CHUS. Written informed consent was obtained from all the participants, and the study was approved by the pertinent ethical committee of the CHUS (ref. 2009/289) and of the INMI (ref 49/2009), IRCCS-CSS (ref 89/2011), UCBM (ref 30/08). 

### 2.2. Expression and Methylation Data from GEO and TCGA Datasets

The analysis of the *B4GALT1* gene was carried out on a multistudy microarray database of CRC expression profiles (total *n* = 1118) based on the Affymetrix U133 Gene Chip microarray platform. Microarray and clinical data were obtained from five GEO public data repositories. Cohort 1: stage I–III CRC patients (*n* = 226, GEO accession number GSE14333) [32]. Cohort 2: stage II–III CRC patients (*n* = 130, GEO accession number GSE37892) [33]. Cohort 3: stage I–IV CRC patients (*n* = 557, GEO accession number GSE39582) [33]. This cohort allowed us to calculate the disease-free survival (DFS), meant as the difference between the time of surgery and the time of the first occurrence of cancer recurrence or death [32,33,34]. Cohort 4: we considered only stage I–III patients (*n* = 125) as done by Lee and colleagues [35]; GEO accession number GSE41258 [36]. We considered the “death” event only if related to cancer disease (cancer-specific survival, CSS). All the other causes of deaths, including unknown causes and living patients were considered “censored” events. Cohort 5: patients with refractory mCRC (*n* = 80, GEO accession number GSE5851) that received cetuximab monotherapy in a clinical trial [37]. For this cohort the progression-free survival (PFS) duration was defined as the time from study enrollment to disease progression or death [38]. Furthermore *KRAS* mutation data were available (exon 2 genomic region). Cohort 6: gene-expression and methylation data were downloaded from Illumina HiSeq RNAseq and Illumina Infinium HumanMethylation450 platforms of The Cancer Genome Atlas [39]. Stage I–IV CRC patients (*n* = 300) were considered excluding mucinous adenocarcinomas. For this study the overall survival (OS), intended as the time from study enrolment to death, was available.

### 2.3. DNA Extraction and Bisulfite Treatment

10 µm sections of FFPE specimens were placed in 1% SDS and proteinase K (Qiagen, Hilden, Germany) at 48 °C overnight followed by DNA extraction with phenol/chloroform and ethanol precipitation. 1 mL of thawed plasma was mixed with 50 µL of 25% SDS and 30 µL of 20 mg/mL proteinase K and incubated at 55 °C for 16 h. After digestion, an equal volume of water-saturated phenol was added into the sample and mixed. Then, the mixture was transferred into a Phase Lock Gel tube (MaXtract High Density tube, Qiagen, Hilden, Germany) followed by centrifugation at 3500× *g* at room temperature for 10 min. The supernatant was transferred into a new tube and mixed with equal volume of chloroform/isoamyl alcohol (24:1) mixture and transferred into a Phase Lock Gel tube followed by centrifugation at 3500× *g* at room temperature for 10 min. 3 mM sodium acetate with a 1:10 ratio of the supernatant and an equal volume of ethanol were added for DNA precipitation at −20 °C overnight. DNA was dissolved in 50 µL of DNA hydration solution (Qiagen, Hilden, Germany) and stored at −20 °C until use. DNA concentration was quantified by the absorbance measurement using the Nanodrop spectrophotometer (Thermo Scientific™, Waltham, MA, USA). DNA extracted from the tumor (2 µg), metastasis (2 µg), and normal colorectal mucosa (2 µg) of a healthy donor and from a cancer patients plasma (500 ng) was subjected to bisulfite treatment and DNA purification using the Epitect Bisulfite kit (Qiagen Sci, MD, Hilden, Germany) according to manufacturer’s instructions.

### 2.4. Quantitative Methylation-Specific PCR (QMSP) and Droplet Digital Quantitative Methylation-Specific PCR (dd-QMSP)

Bisulfite-modified DNA was used as template for quantitative methylation-specific PCR (QMSP) as previously described [31] and droplet digital quantitative methylation-specific PCR (dd-QMSP). For both QMSP and dd-QMSP serial dilutions (250 ng, 25 ng, 2.5 ng, 0.25 ng, 0.025 ng) of CpGenomeTM Universal Methylated DNA (Serologicals Corp., Norcross, GA, USA) were used to construct a calibration curve for the target *(B4GALT1*) and reference (*ACTB*) gene in order to compare the sensitivity of the assays. The 20 µL dd-QMSP reaction included 10 µl of 2X dd-PCR Master Mix (Bio-Rad Laboratories), 1 µL of primers (600 nmol/L, *B4GALT1* Forward 5′-TAGGAAACGGGTTTCGACG-3′, Reverse 5′-CCGTCCACTTTCTTTACCG-3′, *ACTB* Forward 5′-TGGTGATGGAGGAGGTTTAGTAAGT-3′, Reverse 5′-AACCAATAAAACCTACTCCTCCCTTAA-3′), 1 µL of probe (400 nmol/L, *B4GALT1* 5′-FAM-CGTTAAACAACGAAATCCAACCGAA-BHQ-1-3′, *ACTB* 5′-HEX-ACCACCACCCAACACACAATAACAAACACA-BHQ-1-3′), and 5 µL of genomic DNA (gDNA). The dd-QMSP reaction mixture was partitioned into 20,000 water-in-oil droplets in a final volume of 40 µL using the QX1000 Droplet Generator DG8 Cartridge System (Bio-Rad, Pleasanton, CA, USA). The droplets of each sample were transferred in a PCR plate and subjected to end-point PCR on a T100™ (Bio-Rad, Pleasanton, CA, USA). The thermal cycling conditions were 95 °C for 10 min then 40 cycles of 95 °C for 15 s and 56 °C for 1 min with a final 10 min hold at 98 °C. After PCR amplification, each droplet was analyzed in a QX100 Droplet Reader (Bio-Rad, Pleasanton, CA, USA) which assigned each as positive or negative, depending on its fluorescence amplitude. The absolute copy number of the reference (*ACTB*) and target gene (*B4GALT1)* was determined using the Quantasoft 1.7.4 Software (Bio-Rad, Hercules, CA, USA) based on Poisson probability analysis. Each experiment included a positive control sample (CpGenome^TM^ Universal Methylated DNA, Merck Millipore, Billerica, MA, USA) and a negative control (water). To avoid possible biases related to sampling, QMSP and dd-QMSP quantifications were performed on the same diluted gDNA samples loaded in triplicate.

### 2.5. Statistical Analysis

The gene expression and other statistical analyses were performed through R software (ver 3.4.4) Raw data from GEO were downloaded by GEOquery and Biobase packages. TCGA methylation and expression data were downloaded from Wanderer and cBioPortal websites, respectively. For survival analyses on public datasets, patients were dichotomized (High and Low methylation/expression) through the maxstat R package, in order to obtain a significant difference between survival values. The resulting survival curves of dichotomized patients were compared by log-rank tests and plotted as Kaplan–Meier curves. Multivariate Cox proportional hazards regression analysis was used to evaluate the effect of *B4GALT1* expression and methylation on survival, independently of other clinical parameters. In relation to the cohort 5, the Fisher exact test was used to analyze the differences in the response of CRC to cetuximab treatment, whereas the Student *t*-test was applied to verify the expression differences between class members. The nonparametric Spearman’s rank correlation test was used to determine the correlation between the *B4GALT1* mRNA level and the mean methylation values of four CpG sites located within its promoter in the TCGA dataset. Concordance analysis between the primary tumor and matched metastases of continuous methylation data was performed through concordance correlation coefficients. The evaluation of the limit of detection (LOD) between QMSP and dd-QMSP for *B4GALT1* promoter methylation was achieved by using the linear regression model. Patients’ baseline characteristics were reported as the median and interquartile range (IQR) or frequencies and percentages for continuous and categorical variables, respectively. Multiple comparisons between tumor, metastasis, and normal tissue or plasma samples in terms of methylation level were performed with the nonparametric Kruskal–Wallis and Mann–Whitney tests using SPSS 24.0 software (IBMCorp., Armonk, NY, USA). The discriminatory power of methylated *B4GALT1* in plasma samples was assessed by estimating the area under the receiver operating characteristics (ROC) curves, using methylation levels in cancer-free subjects and tumor samples. The optimal cutoff was assessed by jointly maximizing sensitivity and specificity which were reported along with their 95% confidence intervals (CI). The area under the ROC curve (AUC) was also reported as well as its 95% CI. A *p* value < 0.05 was considered statistically significant for all the analyses.

## 3. Results

### 3.1. Study Design and Characteristics of CRC Patient Cohorts

In the present study we included different sample cohorts in order to conduct a comprehensive analysis of the clinical significance of *B4GALT1* (Figure 1). We previously analyzed the expression of *B4GALT1* in primary tumors of CRC cases and a statistically significant inverse correlation was observed between *B4GALT1* methylation status and mRNA expression levels [30,31]. Based on the availability of both expression and methylation data in the TCGA cohort (COAD) we confirmed this correlation (Spearman correlation −0.3018 *p* = 0.0001, Figure S1). We then examined the *B4GALT1* gene expression status in 1418 CRC patients included in six cohorts of public microarray datasets and its correlation with the patient clinical characteristics (Table S2). We found that 11.6% to 80.5% of the patients had a low expression of *B4GALT1* gene in the six cohorts analyzed. Patients with lower expression of the *B4GALT1* gene showed more advanced disease than patients with higher expression of the gene in four out of five cohorts where the stage data were available: cohort 1 (*p* = 0.030), cohort 2 (*p* < 0.0001), cohort 3 (*p* = 0.0086), cohort 4 (*p* = 0.0004), cohort 6 (*p* = 0.608). The study population included also two training and two validation sets in order to evaluate the methylation status of *B4GALT1* in the primary tumor, metastasis, and plasma specimens of mCRC patients (Table S1). Demographics and clinical information related to all selected patients were obtained from hospital records and are summarized in Table S3. The median age of the study population was 68.5 years (range, 42 to 79), 63 years (range, 47 to 84) and 64 years (range, 38 to 93) respectively for UCBM, IRCCS-CSS and CHUS patients. All patients have showed metastases at the time of diagnosis (synchronous metastases) or metachronous events. Table S1 reports an overview of the sample selection. In order to perform the methylation analysis by QMSP of *B4GALT1* in metastatic lesions 27 cases have been used as training set (Group 1: UCBM) and 22 patients from a different institution represented a validation set (Group 2: IRCCS-CSS). Furthermore, we selected a different group of patients including 25 cases consisting of primary tumors and plasma specimens as a training set (Group 3T from CHUS), and an additional 26 cases only with plasmas as a validation set (Group 3V from CHUS). Because of the different sensitivity of the assays used all primary tumors and metastasis specimens were analyzed by QMSP, whereas all plasma samples underwent dd-QMSP.

### 3.2. Downregulated Expression of B4GALT1 is Associated with Poor Survival in Primary Tumors of CRC in GEO and TCGA Dataset

**Figure 2:**
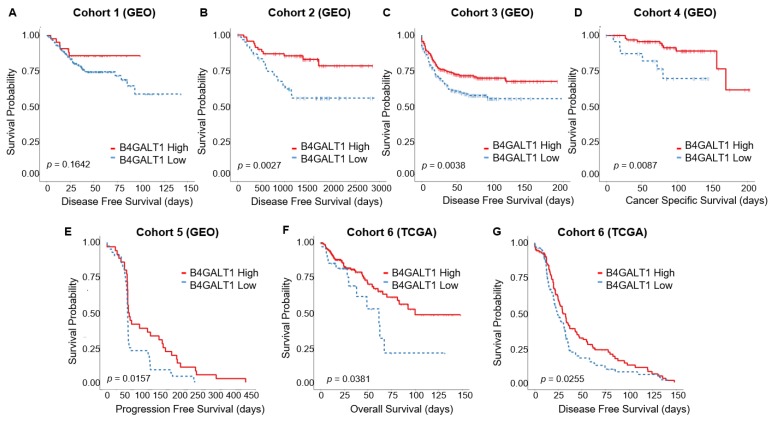
Kaplan–Meier survival curves of *B4GALT1* high (red line) and low (blue line) expression groups in GEO and TCGA cohorts 1–6. (**A**) Disease-free survival (DFS) in GEO Cohort 1 (*n* = 226), (**B**) DFS in GEO Cohort 2 (*n* = 130), (**C**) DFS in GEO Cohort 3 (*n* = 557), (**D**) cancer-specific survival (CSS) in GEO Cohort 4 (*n* = 125), (**E**) progression-free survival (PFS) in GEO Cohort 5 (*n* = 80), (**F**) overall survival (OS) in TCGA Cohort 6, (**G**) DFS in TCGA Cohort 6 (*n* = 300).

The prognostic impact of *B4GALT1* gene expression was analyzed using data of CRC patients with stage I–III (cohorts 1–4). DFS was analyzed for cohorts 1 to 3 and CSS for cohort 4. In three out of four datasets Kaplan–Meier curves showed a significantly worse survival duration in patients with *B4GALT1* low expression than in *B4GALT1* high patients (cohort 1 DFS *p* = 0.164; cohort 2 DFS *p* = 0.002; cohort 3 DFS *p* = 0.003; cohort 4 CSS *p* = 0.008) (Figure 2A–D), indicating that the downregulation of *B4GALT1* gene expression is related to poor prognosis for CRC. These results were confirmed also in cohort 5 (all patients have stage IV CRC) and 6 for which PFS, OS, and DFS data were available (Figure 2E–G). Only cohort 5 patients (*n* = 80) received cetuximab monotherapy. Patients with *B4GALT1* low expression levels showed a shorter PFS duration than did *B4GALT1* high patients (*p* = 0.02; Figure 2E), which may suggest a possible role of *B4GALT1* in influencing the cetuximab responsiveness. We conducted further analyses to determine whether the prognostic impact of the *B4GALT1* expression status is independent of other clinical variables. We pooled the patients of cohorts 1 to 3 (*n* = 853) for univariate and multivariate analyses of factors affecting DFS (Table S4). In the univariate analysis, the *B4GALT1* low expression status is significantly related to worse DFS rates (*p* = 0.0038), but this result is not independent of other clinical variables as shown in the multivariate analysis.

### 3.3. Hypermethylation of B4GALT1 is Associated with Poor Survival in Primary Tumors of CRC in GEO and TCGA Datasets

**Figure 3:**
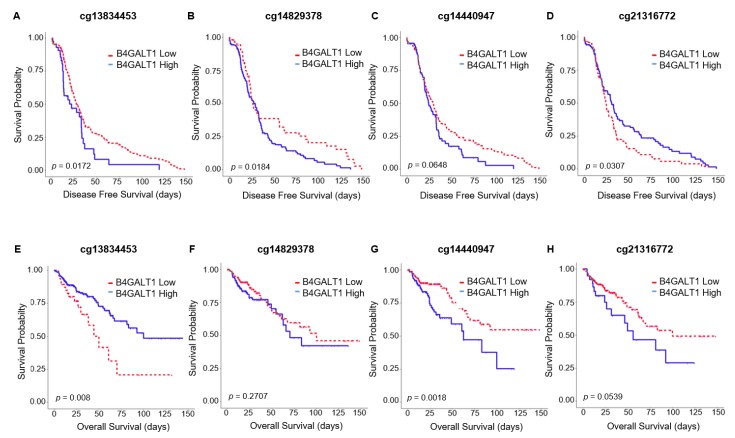
Kaplan–Meier survival curves of high (blue line) and low (red line) methylated *B4GALT1* promoter CpGs in TCGA cohort 6 (*n* = 300). OS in (**A**) cg13834453, (**B**) cg144440947, (**C**) cg14829378, (**D**) cg21316772; DSF in (**E**) cg13834453, (**F**) cg144440947, (**G**) cg14829378, (**H**) cg21316772.

In the patients of the cohort 6 for which also methylation data were available we analyzed four CpG sites and Kaplan–Meier curves showed a correlation between 3/4 methylated CpGs and clinical outcome. Particularly the hypermethylation of cg13834453, cg14829378, and cg14440947 are statistically significantly associated respectively with a worse DFS (cg13834453 *p* = 0.0172; cg14829378 *p* = 0.0184 (Figure 3)) and with a decreased OS (cg14440947 *p* = 0.0018 (Figure 3)). For some CpG (cg21316772, cg13834453) the lack of significance may be due to the methylation data that displayed low and, thus, little informative dynamic ranges.

### 3.4. Downregulated Expression of B4GALT1 is Associated with Cetuximab Resistance in Primary Tumors of CRC in GEO Dataset

**Figure 4:**
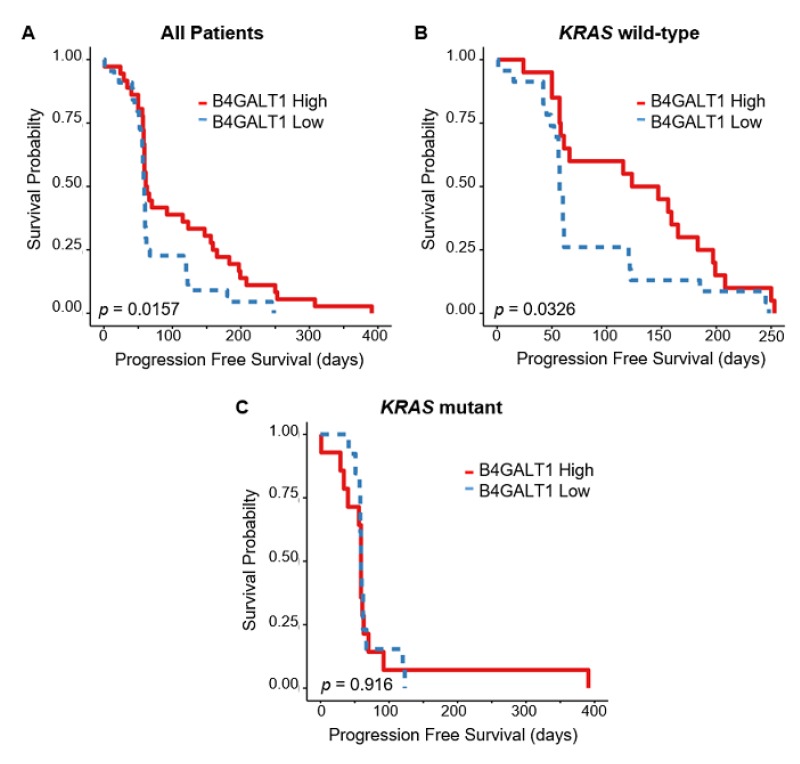
Kaplan–Meier survival curves of *B4GALT1* high (red line) and low (blue line) expression groups in GEO cohort 5 (*n* = 70) according to *KRAS* mutational status. (**A**) PFS in all patients, (**B**) PFS in wild-type (WT)-*KRAS* patients, (**C**) PFS in mutant *KRAS* patients.

The 110 patients of the cohort 5 had mCRC treated with cetuximab monotherapy. For 80/110 cases mRNA expression data were available and 70/80 were also provided *KRAS* mutation status information (Figure 4A). In those cases there was no difference in the *KRAS* mutation rates between the *B4GALT1* high (38.9%) and low (29.5%) expression groups (*p* = 0.665) (Table S2). However we found statistical significant differences in response to Cetuximab between the two groups, where the PFS was lower in patients with downregulation of *B4GALT1* (*p* = 0.01 (Figure 2E)). Interestingly, CRC patients with low expression levels of *B4GALT1* showed indeed a significantly shorter PFS duration than patients with high expression levels of *B4GALT1* and more so in wild-type (WT)-*KRAS* patients (*p* = 0.032 (Figure 4B)) than in *KRAS*-mutant patients (*p* = 0.916 (Figure 4C)). Furthermore, when we restricted the analyses to the group of patients with WT-*KRAS* (Table S5) the disease control rate (partial remission or stable disease) occurred only in patients with *B4GALT1* high expression levels (65%) vs. *B4GALT1* low group patients (30.4%) (*p* = 0.025) (Table S5, panel E).

### 3.5. B4GALT1 Promoter Is Hypermethylated in Metastases of mCRC Patients

**Figure 5:**
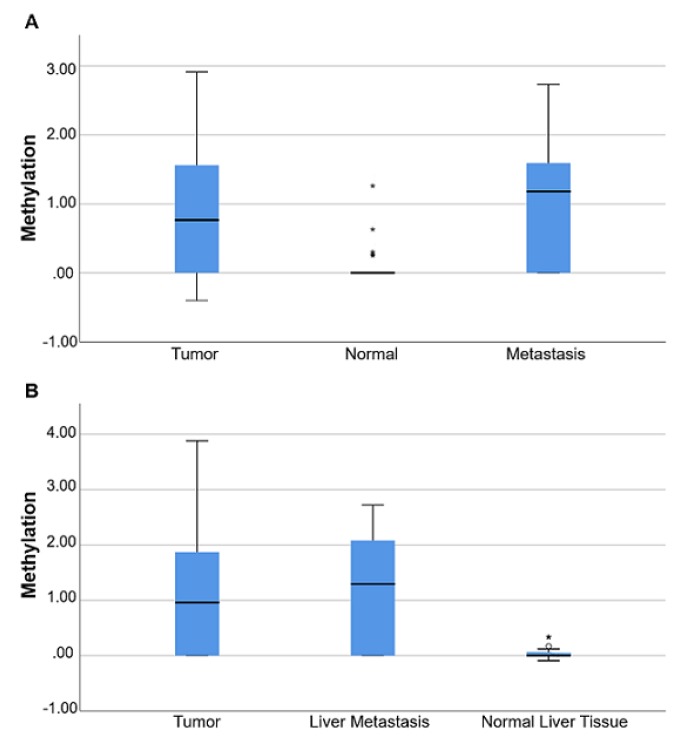
Quantitative methylation-specific PCR (QMSP) analyses of *B4GALT1* in metastatic colorectal cancer (mCRC) tissues. Box plot (logarithmic scale) for *B4GALT1/ACT* ratios determined by QMSP in: (**A**) Training set of tumor samples (*n* = 24) and normal (*n* = 22) and liver metastasis (*n* = 27) (Group 1, Kruskal–Wallis test, *p* = 0.008); (**B**) validation set of tumor samples (*n* = 15) and liver metastasis (*n* = 22) and normal adjacent to the metastasis (*n* = 15) (Group 2, Kruskal–Wallistest, *p* = 0.005). The boxes mark the IQR (interval between the 25th and 75th percentile). The lines inside the boxes denote median values. The whiskers represent the interval between the 10th and 90th percentiles. The asterisks (*) indicate outliers.

The methylation status of the *B4GALT1* promoter was analyzed in an initial training set (Group 1) of 27 CRC patients with liver metastases that underwent surgery at the UCBM. In 24/27 both tumor and metastatic lesions were available; for 3/27 patients only metastasis specimens with no tumors were retrieved; 16/27 metastases were synchronous and 11/27 were metachronous lesions. The methylation analysis was conducted in primary tumors, matched normal colorectal mucosa, and liver metastases. The median value of the *B4GALT1/ACTB* ratio of the two plates was used for statistical analyses. The median values and IQR of *B4GALT1/ACTB* ratios were 0.00 (IQR 0.00–1.92) for normal colorectal mucosa, 4.6 (IQR 0.00–36.23) for primary tumors and 15.24 (IQR 0.00–41.99) for metastatic lesions (*p* = 0.008; Kruskal–Wallis test) (Figure 5A). Furthermore a validation set (Group 2) of 22 mCRC cases containing liver and lung lesions was used and IQR of *B4GALT1/ACTB* ratios were 9.21 (IQR 0.0–297.29) for primary tumors, 24.77 (IQR 0.00–123.13) for metastatic lesions, and 0.00 (IQR 0.00–1.31) for normal parenchyma adjacent metastasis (*p* = 0.005; Kruskal–Wallis test) (Figure 5B). There was no statistically significant difference between the methylation level in liver and lung metastases. Considering the 49 cases of the training (*n* = 27) and validation sets (*n* = 22), both the tumor and matched metastasis were available for 37/49. We considered the methylation as continuous data (concordance correlation coefficient 0.008, 95% CI: 0.3073–0.3225) (Figure S2, panel A) as well as categorical data (Figure S2, panel B). Nineteen out of thirty-seven CRC cases showed methylation alteration both in primary tumors and metastasis tissues. The overall concordance of *B4GALT1* methylation between primary tumor tissues and metastasis samples was 62% (23/37 patients). For 31/37 cases the data related to the timing of metastasis were available. Twenty out of thirty-one were synchronous metastases while 11/31 were metachronous lesions. It is worthy to note that, considering together synchronous and metachronous metastases, 19% of cases (7/37) have shown *B4GALT1* methylation only in the metastases and no methylation in primary tumors. These data suggest that the methylation of this gene could be detected in the circulating compartment even when it is not possible to assess the methylation status of this gene in the primary tumor.

### 3.6. Comparative Analysis of Sensitivity and Specificity of QMSP and dd-QMSP

Given the prognostic role of *B4GALT1* in CRC tissues, we wondered whether this gene could serve as a non-invasive diagnostic biomarker of CRC metastasis. In particular, since the feasibility of methylation detection in plasma rather than downregulation of mRNA, we first assessed the LOD of QMSP and dd-QMSP and we constructed a standard curve with 10-fold serial dilutions of 100% fully-methylated control DNA using water. The LOD of the conventional QMSP was 0.25 ng of DNA per well, which is 800 haploid genome equivalents of methylated *B4GALT1*. In contrast, the LOD of the dd-QMSP assay was 0.025 ng of DNA, which is 80 haploid genome equivalents of methylated *B4GALT1*. The dd-QMSP assay is more sensitive than the conventional QMSP since it was able to detect very low concentrations of templates, 10-fold lower than the QMSP (Figure S3).

### 3.7. Methylation Analysis of B4GALT1 Promoter in Circulating cfDNA of mCRC Patients

**Figure 6:**
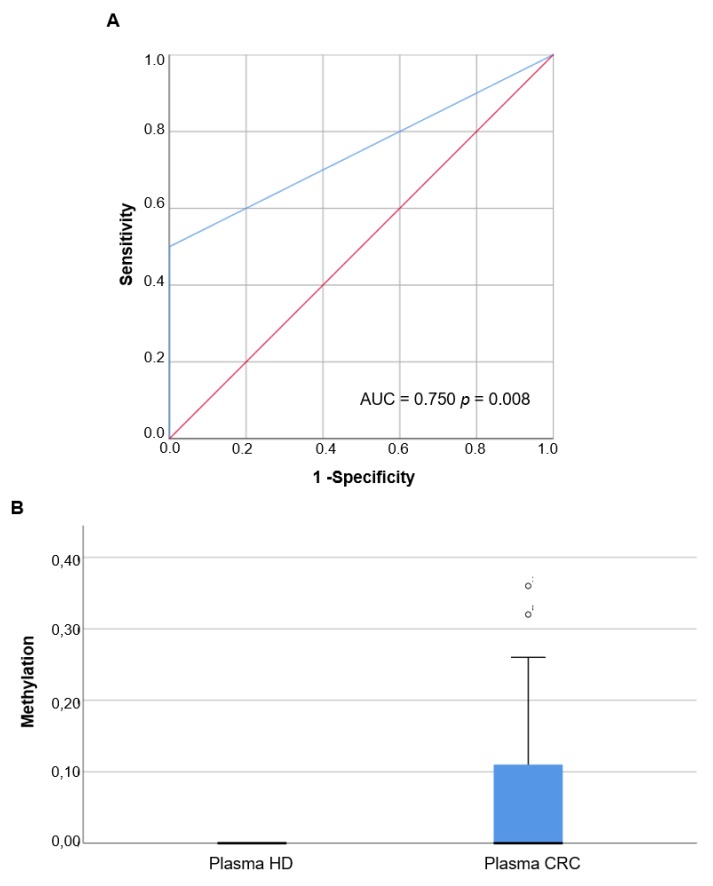
Droplet digital quantitative methylation-specific PCR (dd-QMSP) analyses of *B4GALT1* in mCRC patient plasmas: (**A**) Receiver operating characteristic (ROC) curve analysis in the training set of tumor plasma samples (*n* = 20) and plasma derived from healthy donors (*n* = 19). Area under curve (AUC) = 0.750 (95% CI: 0.592–0.908, *p* = 0.008); (**B**) dd-QMSP in the validation set of tumor plasma samples (*n* = 26) and plasma derived from healthy donors (*n* = 19) (Mann–Whitney test, *p* = 0.001).

Based on the LOD results of QMSP and dd-QMSP, we assessed the discriminatory power of the *B4GALT1* dd-QMSP in 20 plasma samples of mCRC patients obtained from CHUS and 19 healthy control subjects to draw a ROC curve. The AUC value was 0.750 (95% CI: 0.592–0.908, *p* = 0.008) (Figure 6A). Based on the ROC curve, an optimal cut off value of 0.04 was assessed by maximizing the sensitivity and specificity, thereby yielding a 100% specificity and a 50% sensitivity. Methylation of *B4GALT1* showed a highly discriminative ROC curve profile, clearly distinguishing mCRC patients from healthy control subjects. In the training set 9/20 mCRC (45%) displayed *B4GALT1* methylation in primary tumors, among them 5/9 (56%) showed methylation in tumors and plasma as well, whereas 4/9 (44%) cases showed methylation only in the plasma compartment. In a second independent validation set of 26 plasma specimens collected from mCRC patients obtained from CHUS, *B4GALT1* promoter methylation was analyzed only in plasma. The median values and the IQR of *B4GALT1* were 0.00 (IQR 0.00–0.12) for mCRC plasma and 0.00 for plasma derived from healthy subjects (IQR 0.00–0.00) (Mann–Whitney test, *p* = 0.001) (Figure 6B). According to the 0.04 cut-off value derived from the ROC analysis 12/26 (46%) mCRC cases of the validation set showed *B4GALT1* hypermethylation in plasma.

## 4. Discussion

Tumor stage and particularly the presence of metastases are the most critical factors that importantly affect the prognosis of CRC patients. However, the combined use of the most advanced radiologic examinations and the current molecular biomarkers are not entirely reliable to monitor cancer progression and to early detect metastases, so alternative non-invasive tools are urgently needed. Several studies identified novel molecular diagnostic, prognostic, and therapy-response blood-based epigenetic biomarkers, such as *SFRP2, SEPT9, SHOX2,* and *EYA4* [9,14,20].

In previous studies [30,31], we characterized the methylation and expression status of the *B4GALT1* in primary tumors of CRC patients. Here we first confirmed the hypermethylation of *B4GALT1* and its correlation with the expression, taking advantage of the large number of CRC cases of the TCGA dataset. The GEO and TCGA provide precious data to investigate cancer-specific methylated genes due to the large number of well-collected samples [39].

In the six cohorts analyzed (1418 cases) the frequency of downregulated expression of *B4GALT1* ranged from 11.6% to 80.5% of CRC patients. Among these cohorts only five sets provided the data related to the stage. In 4/5 groups the patients with a more advanced disease showed lower expressions of the *B4GALT1* gene, corroborating the hypothesis that the downregulation of the gene may occur more frequently in late phases of cancer progression. 

Patients with low expressions of *B4GALT1* had worse survival durations in terms of DFS and CSS than CRC cases with high expression levels. Nevertheless, in multivariate analysis the low expression status was not significantly related to worse DFS, suggesting that it is not independent of other clinical variables. Furthermore the Kaplan–Meier analysis conducted on the TCGA cohort revealed a correlation between methylated CpGs and clinical outcomes. Indeed, despite the fact that a large number of CpG sites should be analyzed in order to find a panel of significant CpGs that can affect the prognosis [40], we had the chance to analyze a small set of CpGs and we found that 3/4 aberrantly methylated CpGs (cg13834453, cg14829378, and cg14440947) correlate with a worse DFS and OS. The heterogeneous cellular composition of a tumor altogether with the heterogeneous CpG methylation pattern, and the different performances of the analytical platforms available up to date to study the aberrant methylation, are all factors that make the assessment of prognostic significance of these epigenetic modifications very complex due to its multivariable dependency. So a more comprehensively methylation analysis that takes into account a larger number of CpG sites is needed to validate the prognostic impact of *B4GALT1* hypermethylation in CRC. 

Anti-*EGFR* monoclonal antibodies such as cetuximab and panitumumab, used alone or in combination with chemotherapy, are still the standard treatment for mCRC, and they have shown to be more effective in wild-type *RAS* patients. Despite the patient selection based on the pretreatment assessment of *RAS* mutational status, 40–60% are still non-responders [41]. According to the most recent ASCO and ESMO guidelines [42,43], besides mutations of *RAS* family members there are no recommendations for testing additional genes or proteins, suggesting the urgent need to identify additional biomarkers that can be detected in tissue or blood, to predict intrinsic or acquired anti-*EGFR* resistance. Several lines of evidence suggest that post-translational modifications of *EGFR*, such as phosphorylation of the cytoplasmic domain [44] or glycosylation can strongly influence the *EGFR* conformation, its activation [45,46], and the efficiency of antibody-based therapies [47,48], paving the way to explore a wide spectrum of enzymes able to regulate the response to antibody-based drugs. Interestingly, previous works have shown that the ectopic expression of cell surface *B4GALT1* promotes apoptosis in human hepatocellular carcinoma cells inhibiting *EGFR* dimerization and tyrosine phosphorylation, indicating that *B4GALT1* may be an inhibitor of *EGFR* signaling [28,29]. In this perspective in the present study we analyzed the predictive value of *B4GALT1* in a clinical trial of CRC patients [37] treated with anti-*EGFR* in monotherapy, and we showed that the downregulated expression of this gene is significantly associated with poor response to cetuximab therapy, particularly in the CRC cases bearing WT-*KRAS*. Among the selected datasets only TCGA provided methylation data, but due to the lack of cetuximab response data it was not possible to assess the relationship between aberrant *B4GALT1* methylation levels and therapy response. 

Following the investigation of the prognostic and predictive value of the aberrant expression and methylation status of *B4GALT1* conducted in primary tissues of the large GEO and TCGA datasets of CRC patients, we aimed to analyze this gene in metastatic lesions. The analysis of two independent CRC patient cohorts (Group 1 and 2) revealed that *B4GALT1* is significantly hypermethylated in liver and lung metastasis. Interestingly, considering the 37 patients for which primary tumors and matched metastatic lesions were available, 7/37 (19%) cases displayed methylation only in the metastases whereas no *B4GALT1* methylation signal was detected in primary tissues. This finding suggest that the methylation of *B4GALT1* could be detected in circulating compartments even when it is not possible to assess the methylation status of this gene in the primary tumors. This data is also in agreement with other previous works showing that the methylation landscapes of glyco-genes continue to change—and probably during metastatic progression—because tumor cell dissemination requires the interaction between the cell and the surrounding tissue through surface proteins, most of which are glycoproteins [23]. For example, in breast cancer the *GALNT9* gene has been found to be hypomethylated in primary tumors while there is a loss of expression through promoter hypermethylation in brain metastases, thus suggesting a role of *GALNT9* in the later stages of breast cancer [49].

Based on these evidences we assessed the methylation status of *B4GALT1* in plasma specimens of mCRC patients. For this purpose we first compared the performance of two assays, the QMSP and the dd-QMSP, the second of which allowed us to detect a very low concentration of templates, 10-fold lower than the QMSP. By means of dd-QMSP we analyzed hypermethylated *B4GALT1* in plasma specimens of two independent sample cohorts of mCRC cases (Group 3 and 4), showing that the assessment of *B4GALT1* methylation is able to clearly discriminate CRC patients from healthy control subjects (ROC analysis: AUC = 0.750; 95% CI: 0.592–0.908, *p* = 0.008), with a 100% specificity and a 50% sensitivity. This results were confirmed in the validation group were *B4GALT1* is significantly methylated in plasma of cancer patients compared to healthy subjects (Mann–Whitney test, *p* = 0.001). The frequency of *B4GALT1* promoter methylation in plasma, considering both the training and the validation sets, was about 50%. These data mirror the percentage that was found in primary tumors as shown in the present study and also in previous works [30,31]. The strength of this work consist in the stepwise approach of the study design that allowed us to investigate the loss of *B4GALT1,* considering both the expression and methylation status in CRC primary tumors, metastases, and blood compartments in a single study exploring its diagnostic, prognostic, and anti-*EGFR* therapy-predictive significance. Moreover the analyses were carried out including 10 different independent patient cohorts. Nevertheless this study had some limitations that should be addressed. Particularly, in relation to the ability of downregulated expressions of *B4GALT1* to predict cetuximab resistance, we had the chance to conduct the analysis in a clinical trial in which cetuximab was used as monotherapy [37], so both *B4GALT1* downregulated expression and hypermethylation should be assessed in other clinical trials which include the use of cetuximab or panitumumab in combination with chemotherapy. Despite the large size of primary tumor and metastasis tissues analyzed, we had the chance to assess the *B4GALT1* methylation status in only 49 plasma samples. Additional studies that include a larger set of plasma samples are needed to validate the specificity and the sensitivity of hypermethylated *B4GALT1* as circulating biomarkers and to explore the value of the plasma detection of *B4GALT1* in anti-*EGFR* therapy treated CRC in order to predict acquired resistance. 

## 5. Conclusions

This study provide preliminary evidences that the loss of *B4GALT1*, through respectively hypermethylation and downregulated expression, may be a novel prognostic and predictive biomarker useful to identify WT-*KRAS* mCRC patients that may benefit from cetuximab treatment. Nevertheless other confirmatory studies in larger and independent sample cohorts will be needed. Further, the *B4GALT1* promoter hypermethylation occured in primary tumors as well as in metastasis, and it can be a valuable specific and sensitive diagnostic biomarker that can be assessed in the plasma cfDNA of mCRC.

## Supplementary Materials

The following are available online at https://www.mdpi.com/2072-6694/11/10/1598/s1, Table S1: Patients and samples selection of metastatic colorectal cancer, Table S2: Clinical characteristics of 1418 patients in cohort 1 to 6 stratified by expression level of *B4GALT1*, Table S3: Demographic and clinicopathological characteristics of colorectal cancer cases, Table S4: Univariate and multivariate analysis of factors affecting DFS in stage I–III patients, Table S5: *KRAS* mutational status and response to cetuximab chemotherapy according to *B4GALT1* expression status in GEO cohort 5, Figure S1: Inverse correlation of *B4GALT1* mRNA expression with the DNA methylation status in cohort 6 (TCGA-COAD), Figure S2: Concordance analysis between tumor and matched metastases in CRC (Group 1 and 2, *n* = 37), Figure S3: Comparative linear regression analysis of limit of detection (LOD) for *B4GALT1* promoter methylation between dd-QMSP and QMSP.
