# Supplementary material for: Diagnostic and Prognostic Value of B4GALT1 Hypermethylation and Its Clinical Significance as a Novel Circulating Cell-Free DNA Biomarker in Colorectal Cancer
Source: Cancers (Basel). 2019 Oct 19;11(10):1598. doi: 10.3390/cancers11101598 (PMC6826707; doi:10.3390/cancers11101598)
Supplement: Supplementary file 1 [file cancers-11-01598-s001.pdf]

Supplementary Materials: Diagnostic and prognostic value of *B4GALT1* hypermethylation and its clinical significance as a novel circulating cell-free DNA biomarker in colorectal cancer

Francesco Picardo, Antonella Romanelli, Laura Muinelo-Romay, Tommaso Mazza, Caterina Fusilli, Paola Parrella, Jorge Barbazán, Rafael Lopez-López, Raffaela Barbano, Mariangela De Robertis, Chiara Taffon, Veronica Bordoni, Chiara Agrati, Manuela Costantini, Francesca Ricci, Paolo Graziano, Evaristo Maiello, Lucia Anna Muscarella, Vito Michele Fazio and Maria Luana Poeta

Table S1. Patients and samples selection of metastatic colorectal cancer.

Type of Specimens	Group 1 [a] (Training Set)	Group 2 [b] (Validation Set)	Group 3T [c] (Training Set)	Group 3V [c] (Validation Set)	Group 4 [d] (Controls)
Patients	27	22	25	26	19
Tumor Tissue	24/27	15/22	25/25		
Normal Tissue	22/27				
Metastasis Tissue	27/27	22/22	1/25		
Normal tissue surrounding the metastasis		15/22			
Plasma			20/25	26/26	19/19
Analysis performed	QMSP [e]	QMSP	QMSP + dd-QMSP [f]	dd-QMSP	dd-QMSP

[a] Group 1: metastatic colorectal cancer patients from University Campus Bio-Medico of Rome (UCBM). [b] Group 2: metastatic colorectal cancer patients from "Ospedale Casa Sollievo della Sofferenza" (IRCCS-CSS). [c] Group 3T and 3V: metastatic colorectal cancer patients from University Hospital of Santiago de Compostela (CHUS). [d] Group 4: healthy controls from the "Lazzaro Spallanzani" National Institute for infectious Diseases (INMI) of Rome, and from University Hospital of Santiago de Compostela (CHUS). [e] QMSP: Quantitative-Methylation-Specific-PCR. [f] dd-QMSP: Droplet-Digital-Quantitative-Methylation-Specific-PCR.

Table S2. Clinical characteristics of 1418 patients in cohort 1 to.6 stratified by expression level of *B4GALT1*.

Cohort 1 (*n* = 226)			
Variable	*B4GALT1* low	*B4GALT1* high	*p*-value
Gender [a]	*N* = 182 (80.5%)	*N* = 44 (19.5%)	0.6471
Male	98 (53.8%)	22 (50%)	
Female	84 (46.2%)	22 (50%)	
Age (mean ± SD) [b]	65.72 ± 13.35	67.32 ± 11.58	0.4653
Stage [a]	*N* = 182 (80.5%)	*N* = 44 (19.5%)	0.0300
I	31 (17%)	10 (22.7%)	
II	70 (38.5%)	24 (54.5%)	
III	81 (44.5%)	10 (22.7%)	
Location [a]	*N* = 180 (80.4%)	*N* = 44 (19.6%)	0.0874
Proximal R	75 (41.7%)	26 (59.1%)	
Distal L	78 (43.3%)	15 (34.1%)	
Rectum	27 (15%)	3 (6.8%)	
Cohort 2 (*n* = 130)			

Gender [a]	*N* = 60 (46.2%)	*N* = 70 (53.8%)	0.9569
Male	32 (53.3%)	37 (52.9%)	
Female	28 (46.7%)	33 (47.1%)	
Age (mean ± SD) [b]	69.28 ± 12.69	67.37 ± 12.71	0.3938
Stage [a]	*N* = 60 (46.2%)	*N* = 70 (53.8%)	<0.0001
I	0 (0%)	0 (0%)	
II	20 (33.3%)	53 (75.7%)	
III	40 (66.7%)	17 (24.3%)	
Location [a]	*N* = 59 (45.7%)	*N* = 70 (54.3%)	0,7045
Proximal R	25 (42.4%)	32 (45.7%)	
Distal L	34 (57.6%)	38 (54.3%)	
Rectum	0 (0%)	0 (0%)	
Cohort 3 (*n* = 557)			
Gender [a]	*N* = 206 (37%)	*N* = 351 (63%)	0.2548
Male	120 (58.3%)	187 (53.3%)	
Female	86 (41.7%)	164 (46.7%)	
Age (mean ± SD)[b]	66.49 ± 13.35	67.06 ± 13.28	0.6276
Stage[a]	*N* = 206 (37%)	*N* = 351 (63%)	0.0086
I	13 (6.3%)	23 (6.6%)	
II	78 (39.7%)	182 (51.9%)	
III	86 (41.7%)	115 (32.8%)	
IV	29 (14.1%)	31 (8.8%)	
Location [a]	*N* = 206 (37%)	*N* = 351 (63%)	0.0015
Proximal R	63 (30.6%)	155 (44.2%)	
Distal L	143 (69.4%)	196 (55.8%)	
Rectum	0 (0%)	0 (0%)	
KRAS status [a]	*N* = 194 (36.2%)	*N* = 342 (63.8%)	0.0532
Wild type	106 (51.5%)	216 (61.5%)	
Mutant	88 (42.7%)	126 (35.9%)	
Cohort 4 (*n* = 125)			
Gender [a]	*N* = 23 (18.4%)	*N* = 102 (81.6%)	0.2333
Male	9 (39.1%)	54 (52.9%)	
Female	14 (60.9%)	48 (47.1%)	
Age (mean ± SD) [b]	64.26 ± 12.48	65.02 ± 13.75	0.8084
Stage [a]	*N* = 23 (18.4%)	*N* = 102 (81.6%)	0.0004
I	4 (17.4%)	24 (23.5%)	
II	2 (8.7%)	46 (45.1%)	
III	17 (73.9%)	32 (31.4%)	
IV	0 (0%)	0 (0%)	
Location [a]	*N* = 23 (18.4%)	*N* = 102 (81.6%)	0.8523
Proximal R	8 (34.8%)	42 (41.2%)	
Distal L	13 (56.5%)	52 (51%)	
Rectum	2 (8.7%)	8 (7.8%)	
Cohort 5 (*n* = 80)			
Gender [a]	*N* = 44 (55%)	*N* = 36 (45%)	0.3234
Male	22 (50%)	22 (61.1%)	
Female	22 (50%)	14 (38.9%)	
Age (mean ± SD) [b]	61.26 ± 12.33	60.39 ± 14.36	0.7734
KRAS status [a]	*N* = 36 (51.4%)	*N* = 34 (48.6%)	0.6657
WT	23 (52.3%)	20 (55.6%)	
Mutant	13 (29.5%)	14 (38.9%)	
Cohort 6 TCGA (*n* = 300)			
Gender [a]	*N* = 35 (11.6%)	*N* = 265 (88.4%)	0.4673
Male	17 (48.6%)	146 (55.1%)	
Female	18 (51.4%)	119 (44.9%)	
Age (mean ± SD) [b]	67.23 ± 13.45	65.19 ± 13.07	0.3888
Stage [a]	*N* = 34 (11.3%)	*N* = 256 (88.7%)	0.6082
I	3 (8.6%)	46 (17.2%)	

II	15 (42.9%)	103 (38.6%)	
III	11 (31.4%)	76 (28.5%)	
IV	5 (14.3%)	31 (11.6%)	
KRAS status [a]	*N* = 6 (2%)	*n* = 33 (98%)	0.8393
WT	3 (8.6%)	18 (6.7%)	
Mutant	3 (8.6%)	15 (5.6%)	

[a] χ^2-test. [b] *t*-test.

Table S3. Demographic and clinicopathological characteristics of colorectal cancer cases

Variable	Group 1	Group 2	Group 3T+ 3V
Total Patients	27/27	8/22 [a]	54/54
Gender			
Male	19 (70,4%)	7 (87,5%)	17 (31,4%)
Female	8 (29,6%)	1 (12,5%)	37 (68,5%)
Age			
Median (Range)	68,5 (42–79)	63 (47–84)	64 (38–93)
Tumor site			
Rectum	3 (11,1%)	2 (25%)	18 (33,3%)
Sigmoid colon	2 (7,4%)	2 (25%)	19 (35,1%)
Descending colon	5 (18,5%)	1 (12,5%)	1 (1,8%)
Transverse colon	0 (0%)	0 (0%)	7 (12,9%)
Ascending colon	17 (63%)	3 (37,5%)	9 (16,6%)
Histologic type			
Adenocarcinoma moderately differentiated	25 (92,6%)	8 (100%)	53 (98,1%)
Infiltrating adenocarcinoma	2 (7,4%)	0 (0%)	1 (1,9%)
Grading			
G1	0 (0%)	0 (0%)	14 (25,9%)
G2	21 (77,8%)	3 (37,5%)	22 (40,7%)
G3	5 (18,5%)	0 (0%)	6 (11,1%)
undetermined	1 (3,7%)	5 (62,5%)	12 (22,2%)
Pathologic tumor stage			
T1	1 (3,7%)	0 (0%)	0 (0%)
T2	2 (7,4%)	0 (0%)	2 (3,7%)
T3	17 (63%)	2 (25%)	30 (55,5%)
T4	7 (25,9%)	0 (0%)	11 (20,3%)
X		6 (75%)	11 (20,3%)
Pathologic nodal stage			
n+	18 (66,7%)	1 (12,5%)	35 (64,8%)
n−	9 (33,3%)	3 (37,5%)	5 (9,2%)
X		4 (50%)	14 (25,9%)
Site of Metastasis [b]			
Liver	27 (90%)	4 (40%)	43 (55,8%)
Lung	0 (0%)	2 (20%)	17 (22%)
Peritoneum	1 (3,3%)	1 (10%)	8 (10,3%)
Other	2 (6,7%)	1 (10%)	9 (11,6%)
UICC stage [c]			
I	2 (7,4%)	0 (0%)	0 (0%)
II A	3 (11,1%)	2 (25%)	0 (0%)
II B	0 (0%)	0 (0%)	0 (0%)
III A	1 (3,7%)	0 (0%)	0 (0%)
III B	0 (0%)	1 (12,5%)	0 (0%)
III C	2 (7,4%)	0 (0%)	0 (0%)
IV	19 (70,4%)	5 (62,5%)	54 (93%)
Alcohol			
No	0 (0%)	0 (0%)	7 (12,9%)
Yes	0 (0%)	0 (0%)	10 (18,5%)

undetermined	27 (100%)	8 (100%)	37 (68,5%)
Smoke			
No	0 (0%)	0 (0%)	11 (20,3%)
Yes	0 (0%)	0 (0%)	10 (18,5%)
undetermined	27 (100%)	8 (100%)	33 (61,1%)
KRAS mutation			
WT [d]	0 (0%)	4 (50%)	30 (55,5%)
Codon 12/13 exon 2	0 (0%)	0 (0%)	5 (9,2%)
Codon 12	1 (3,7%)	3 (37,5%)	8 (14,8%)
Codon 13	0 (0%)	1 (12,5%)	5 (9,2%)
Codon 61	0 (0%)	0 (0%)	1 (1,8%)
undetermined	26 (96,3%)	0 (0%)	5 (9,2%)
BRAF mutation			
WT [d]	1 (3,7%)	0 (0%)	1 (1,9%)
V600E exon 15	0 (0%)	0 (0%)	0 (0%)
undetermined	26 (96,3%)	8 (100%)	53 (98,1%)
MSI status			
MSS	0 (0%)	0 (0%)	7 (13%)
MSI	0 (0%)	0 (0%)	0 (0%)
undetermined	27 (100%)	8 (100%)	47 (87%)

[a] Group 2: the Demographic and clinicopathological information were available for 8/22 cases. [b] Some patient had more than one metastasis at different sites. [c] UICC: Union for International Cancer Control. [d] WT: wild type.

Table S4. Univariate and multivariate analysis of factors affecting DFS in stage I-III patients (data from GEO cohorts 1, 2 and 3 were pooled together n = 853).

		**Univariate analysis [a]**		**Multivariate analysis [b]**	
**Variables**	***n***	**5-Years DFS**	***p*-value**	**HR (95% CI)**	***p*-value**
<70 y	447	68.50%	-	-	-
≥70 y	405	74.60%	0.1517	-	-
Female	388	75.20%	0.0760	0.7725 (0.57–1.03)	0. 0835
Male	465	68.00%	-	1 (-)	-
Left	462	69.30%	-	-	-
Rectum	30	77.50%	-	-	-
Right	358	73.30%	-	-	-
Done	289	64.40%	-	1.0133 (0.72–1.42)	0.9395
Undone	433	77.30%	-	1 (-)	-
I	77	95.40%	-	0.1758 (0.05–0.55)	0.0033
II	427	79.10%	-	1 (-)	-
III	349	57.10%	-	2.0515 (1.45–2.90)	0.0001
High	434	75.80%	0.0038	1 (-)	-
Low	419	66.50%	-	1.2133 (0.91–1.62)	0.1938

[a] In univariate analyses, log-rank tests were conducted. [b] In the multivariate Cox proportional hazard model, only variables with $p < 0.15$ in univariate analysis were included and the "enter method" was applied. [c] Data on age of GSM972293 of cohort 3 was not specified.

Table S5. *KRAS* mutational status and response to cetuximab monotherapy according to *B4GALT1* expression status in GEO cohort 5 (n = 80).

(**A**) Disease control rate versus *B4GALT1* expression (n = 80; p = 0.0708)		
Heading Title	*B4GALT1* High (36)	*B4GALT1* Low (44)
CR/PR/SD	15 (41.7%)	10 (22.7 %)
PD/UTD	21 (58.3%)	34 (77.3%)

All 80 patients were included in this analysis. Abbreviations: CR, complete remission; PR, partial remission; SD, stable disease; PD, progressive disease, UTD, undetermined.

(**B**) Disease control rate versus *B4GALT1* expression (n = 68/80; p = 0.1056)		
Heading Title	*B4GALT1* High (32)	*B4GALT1* Low (36)
CR/PR/SD	15 (46.9%)	10 (27.8%)
PD	17 (53.1%)	26 (72.2%)

68/80 patients were included and 12/80 patients without response data (UTD, undetermined) were excluded in this analysis. Abbreviations: CR, complete remission; PR, partial remission; SD, stable disease; PD, progressive disease.

(**C**) Response rate versus *B4GALT1* expression (in *KRAS* WT patients n = 43/80; p = 0.5250)		
Heading Title	*B4GALT1* High (20)	*B4GALT1* Low (23)
PR	3 (15%)	2 (8.7 %)
SD/PD/UTD	17 (85%)	21 (91.3%)

43/80 patients with *KRAS* WT were included in this analysis. Abbreviations: PR, partial remission; SD, stable disease; PD, progressive disease, UTD, undetermined; WT, wild-type.

(**D**) Response rate versus *B4GALT1* expression (in *KRAS* WT patients n = 39/80; p = 0.680)		
Heading Title	*B4GALT1* High (20)	*B4GALT1* Low (19)
PR	3 (15%)	2 (10.5 %)
SD/PD	17 (85%)	17 (89.5%)

39/80 patients with *KRAS* WT were included in this analysis, 4/80 patients with *KRAS* WT who had no response data (UTD) were excluded. Abbreviations: PR, partial remission; SD, stable disease; PD, progressive disease, WT, wild-type.

(**E**) Disease control rate versus *B4GALT1* expression (in *KRAS* WT patients n= 43/80; p = 0.0251)		
Heading Title	*B4GALT1* High (20)	*B4GALT1* Low (23)
PR/SD	13 (65%)	7 (30.4 %)
PD/UTD	7 (35%)	16 (69.6%)

43/80 patients with *KRAS* WT were included in this analysis. Abbreviations: PR, partial remission; SD, stable disease; PD, progressive disease; UTD, undetermined WT, wild-type.

(**F**) Disease control rate versus *B4GALT1* expression (in *KRAS* WT patients n = 39; p = 0.0826)		
Heading Title	*B4GALT1* High (20)	*B4GALT1* Low (19)
PR/SD	13 (65%)	7 (36.8%)
PD	7 (35%)	12 (63.2%)

39/80 patients with *KRAS* WT were included in this analysis, 4/80 patients with *KRAS* WT who had no response data (UTD) were excluded. Abbreviations: PR, partial remission; SD, stable disease; PD, progressive disease, WT, wild-type.

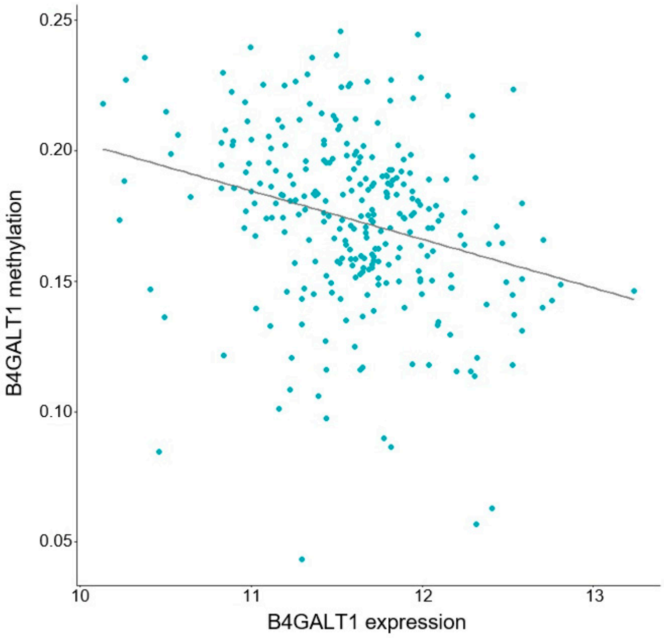

Figure 1. Inverse correlation of *B4GALT1* mRNA expression with the DNA methylation status in cohort 6 (TCGA-COAD). Spearman correlation coefficient = −0.3018, $p = 0.0001$.

A

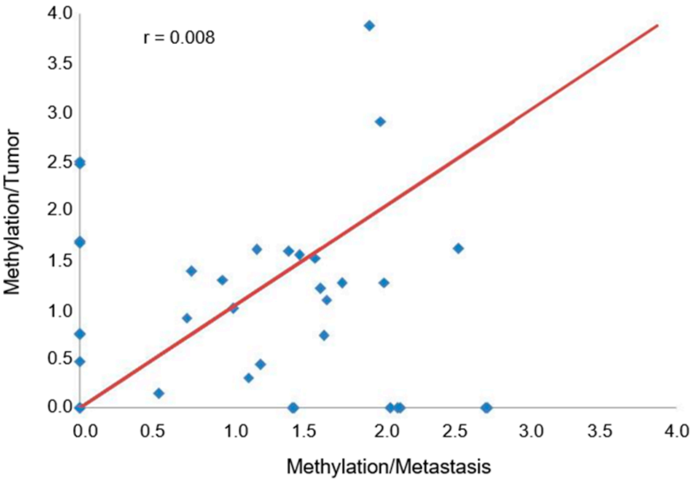

B

	Metastasis Methylation		
**Tumor Methylation**	**Methylated**	**Unmethylated**	**Total**
**Methylated**	19/37 (51%)	7/37 (19%)	26 (70%)
**Unmethylated**	7/37 (19%)	4/37 (11%)	11 (30%)
**Total**	26 (70%)	11 (30%)	37 (100%)

Figure 2. Concordance analysis between tumor and matched metastases in CRC (Group 1 and 2, n = 37). (**A**) The concordance analysis was conducted considering the methylation continuous data. Concordance correlation coefficient (r) = 0.008 ($r \leq 0.90$ indicates poor degree of concordance; r = 0.90–0.95 indicates moderate degree of concordance; r = 0.95–0.99 indicates substantial degree of concordance; $r > 0.99$ indicates almost perfect degree of concordance). (**B**) The concordance analysis was conducted considering the methylation status as categorical data based on the cut-off defined by the presence of methylation in normal colon tissue. Concordance correlation coefficient (r) = 0.095 ($r \leq 0.20$ indicates poor degree of concordance; r = 0.21–0.40 indicates fair degree of concordance; r = 0.41–0.60 indicates moderate degree of concordance; r = 0.61–0.80 indicates good degree of concordance; r = 0.81–1 indicates very good degree of concordance).

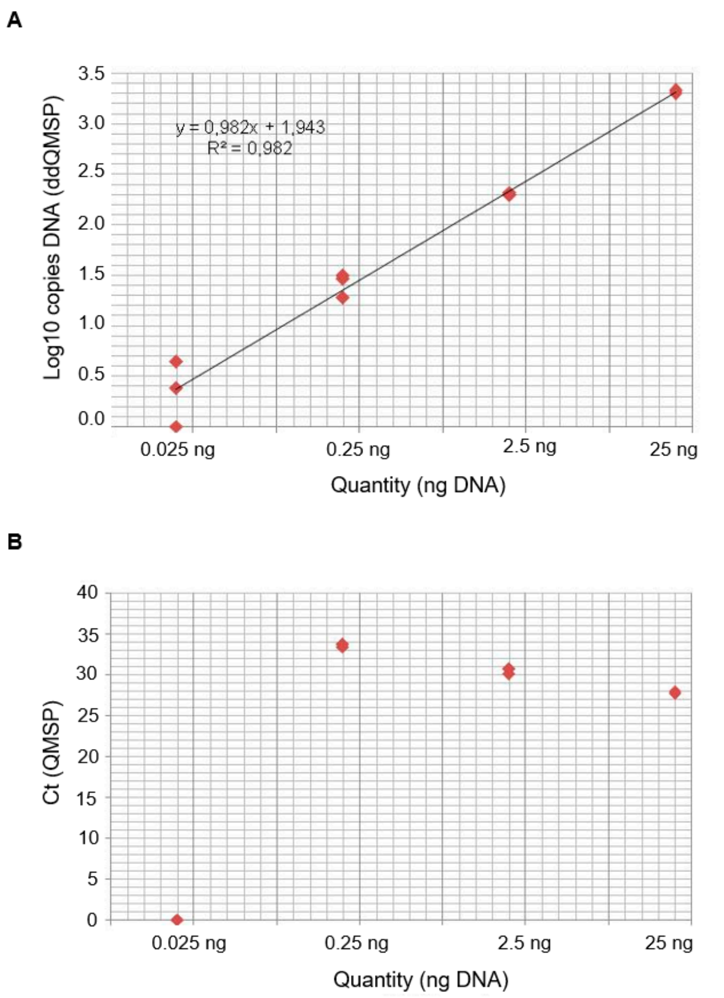

Figure S3: Comparative Linear Regression analysis of limit of detection (LOD) for *B4GALT1* promoter methylation between dd-QMSP and QMSP. For both assays 10-fold serial dilutions (250ng, 25ng, 2.5ng, 0.25ng, 0.025 ng) of 100% Methylated Control DNA were used to construct a calibration curve. (**A**) The log10-transformed 100% methylated DNA copy numbers determined by dd-QMSP were plotted against the corresponding standard curve dilutions (**B**) The ct values of 100% methylated DNA determined by QMSP were plotted against the corresponding standard curve dilutions.

© 2019 by the authors. Licensee MDPI, Basel, Switzerland. This article is an open access article distributed under the terms and conditions of the Creative Commons Attribution (CC BY) license (http://creativecommons.org/licenses/by/4.0/).
